# Correlating advanced microscopies reveals atomic-scale mechanisms limiting lithium-ion battery lifetime

**DOI:** 10.1038/s41467-021-24121-9

**Published:** 2021-06-18

**Authors:** Baptiste Gault, Jonathan D. Poplawsky

**Affiliations:** 1 Max-Planck-Institut für Eisenforschung, Düsseldorf, Germany; 2 Department of Materials, Royal School of Mines, Imperial College, London, UK; 3 Center for Nanophase Materials Sciences, Oak Ridge National Laboratory, Oak Ridge, TN USA

**Keywords:** Energy, Batteries, Structural properties, Characterization and analytical techniques

## Abstract

The longevity of a lithium-ion battery is limited by cathode degradation. Combining atom probe tomography and scanning transmission electron microscopy reveals that the degradation results from atomic-scale irreversible structural changes once lithium leaves the cathode during charging, thereby inhibiting lithium intercalation back into the cathode as the battery discharges. This information unveils possible routes for improving the lifetime of lithium-ion batteries.

Lithium-ion batteries have received great attention due to their widespread use for applications ranging from small electronic devices, for instance mobile phones, to high-capacity energy storage, such as in electric vehicles and grid energy. A major limiting factor in renewable energy grid integration is the ability to reliably store and access electricity at a low cost. For example, solar panels are economically competitive with fossil fuels; however, solar energy is produced at nature’s will and must be stored for future distribution in the event of solar power surpluses. Li-ion batteries are among the best commercially viable energy storage technologies, but unfortunately, capacity degradation limits their lifetime and battery replacement is expensive. Engineering Li-ion batteries to extend their operational lifetime will decrease cost and limit the environmental footprint associated with renewable energy compared to other energy sources, facilitating widespread usage^1^. Understanding capacity degradation mechanisms is necessary to rationally design new materials or implement operational protocols to enhance durability, but degradation mechanisms are complex and typically occur across multiple length scales, some of which are not readily accessible without using high spatial resolution characterization techniques.

Li-ion batteries consist of a cathode/electrolyte/anode heterostructure, as shown in Fig. 1. Li-ions leave the oxidized cathode host and are then inserted within or electrodeposited onto the reduced anode during charging and then collect back in the cathode via a reduction reaction as the battery discharges^2,3^. High-energy density Li-ion batteries require high-voltage cathodes that are typically an oxide compound containing Li and at least one transition metal (Co, Ni, etc.)^4^. In fact, the discovery of Co-based oxides for use as Li-ion battery cathodes led to the Nobel Prize in Chemistry in 2019 to early pioneers Goodenough, Whittingham, and Yoshino^5^.

**Fig. 1:**
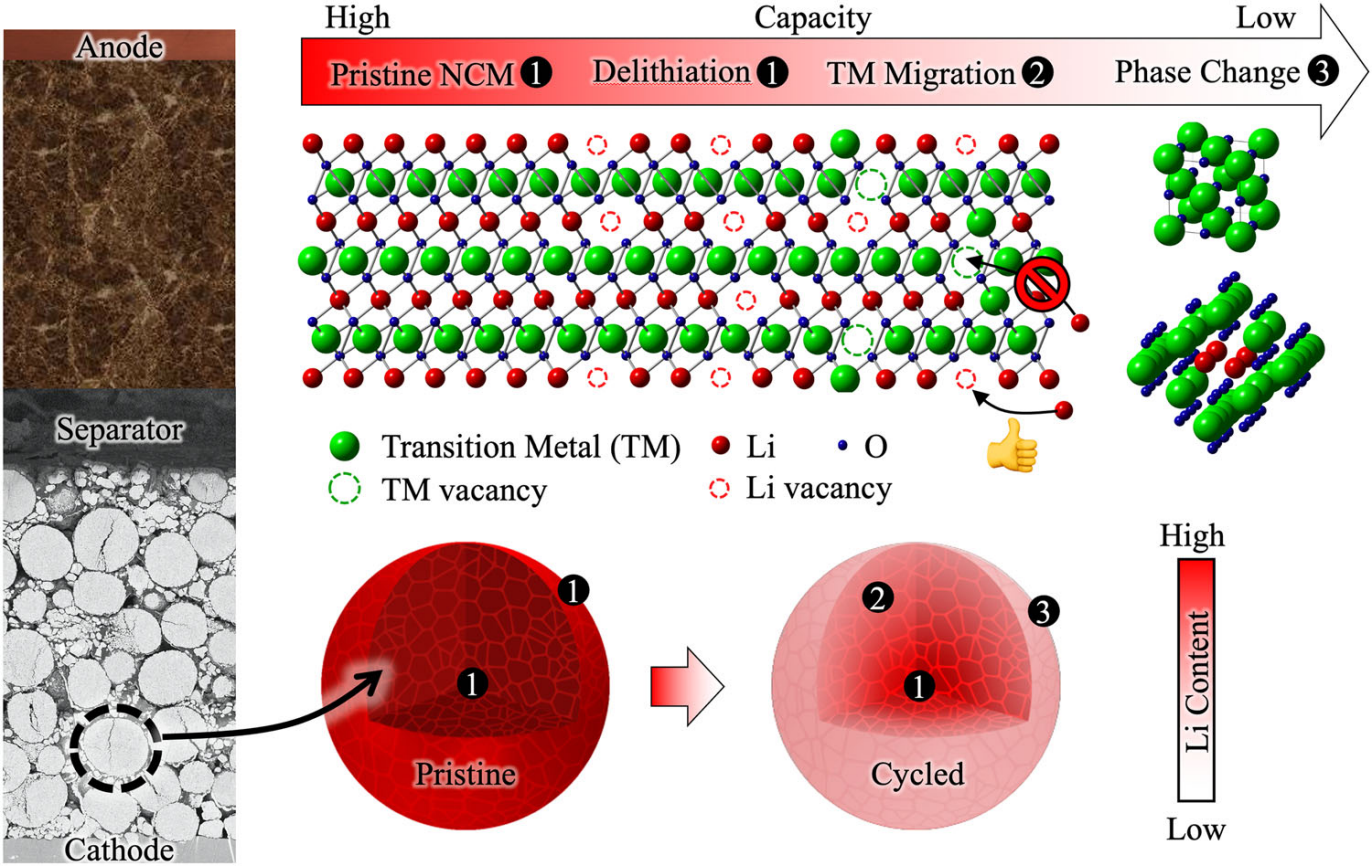
(Left) Cross-section schematic of a Li ion battery with a superimposed scanning electron microscope image of the cathode material studied by Chae et al. composed of ~10 µm NCM particles. After cycling, Li is lost from the cathode and NCM particles exhibit a graded Li concentration with a pristine Li content in the center and an almost zero Li concentration at the near surface region. (top) As the battery is cycled, TMs migrate to Li sites during charging blocking Li ions from returning to the structure, which reduces capacity. A phase change into rock salt- and spinel-like structures occurs after severe TM migration, further reducing the capacity.

Li-ion battery capacity degradation originates from structural defects that are created by compositional instabilities in the high-voltage cathode. Lithium removal from metal oxides at high voltage causes structural changes, which can result in defect formation including anti-site defects. Successive charge-discharge cycles lead to defect accumulation that embrittles the cathode and drives capacity loss^6–8^. Correlating the Li distribution and atomic structure at the relevant length scales over the lifetime of a battery is crucial to unlocking the degradation processes and provide the insight needed to limit or avoid it.

(Scanning) transmission electron microscopy ((S)TEM) techniques and atom probe tomography (APT), separately or in combination, are commonly used to characterize the structure and chemistry of materials. STEM provides a two-dimensional, projected image of the material and can attain sub-Å spatial resolution, revealing the structural organization of the atoms, which can also be chemically identified using spectroscopy techniques such as X-ray energy dispersive spectroscopy (EDS) or electron energy loss spectroscopy (EELS). Additionally, electron diffraction can identify the crystallographic phases at the nanoscale^9^. However, Li-containing materials are typically unstable under electron beam irradiation, and the detectability and quantification of light elements is challenging. APT is a time-of-flight mass spectrometer combined with a three-dimensional projection microscope having sub-nm spatial resolution. Aberrations in the projected ion trajectories limit the ability of APT to extract structure or elemental site occupancy, but its compositional sensitivity, in the tens of parts-per-million, extends to light elements, including Li, albeit not without challenges^10–12^. Fortunately, the shortcomings of either technique are complemented by the strengths of the other technique^13^ and therefore, a combined APT and STEM experiment can enable the correlation of atomic structure with nanoscale Li concentrations.

To better understand the creation of the observed Li-ion concentration gradients and track the chemical evolution during electrochemical cycling, Chae et al. combined the strengths of STEM and APT to probe the chemical and compositional changes of a Li(Ni_0.80_Co_0.15_Mn_0.05_)O_2_ (NCM)-based cathode^14^. The cathode is an agglomeration of approx. 10 µm “powder grains” that each contain several 100 nm-sized particles sintered together, such that only the surface of the 10 µm particles contact the electrolyte. Inductively coupled plasma-atomic emission spectroscopy (ICP-AES), a bulk composition measurement technique, confirms the loss of Li within the cathode over the course of multiple cycles, but the spatial resolution of this method is simply insufficient to determine the origin of the Li loss.

Chae et al. prepared specimens for STEM and APT studies at different depths from the surface of NCM cathode particles to assess their structural and compositional evolution after cycling. The NCM particles had a Li:Ni:Co:Mn = 1.04:0.80:0.15:0.05 in the pristine state. The researchers found that the Li content was significantly reduced after cycling and a radial gradient was observed in the Li content, i.e., the center of the particle retained the pristine Li content while the particle surface had the lowest Li content. The gradient and Li-loss were found to increase after subsequent charge-discharge cycles. STEM imaging revealed structural defects, i.e. dislocations and anti-phase domain boundaries, and a phase change associated with the migration of TMs into Li-sites, similar to previously published results^15^. APT revealed an inhomogeneous distribution of Li ions and Li-enrichment associated with the structural defects observed by STEM. The number density of such defects is low, and therefore, these defects likely do not significantly impact capacity degradation.

What makes this work unique is the combined APT/STEM approach revealing a direct link between the amount of Li-depletion in the NCM and the degree of structural disorder. As the cathode delithiates during charging, the TM ions move into the more energetically favorable Li sites^15–17^ as there is little to no repulsion from neighboring cationic species, while Li cannot readily occupy the TM vacancies on their return during discharge cycles. Significant TM ion migration into Li sites facilitates a phase change to rock salt or spinel-like structures, which inhibits subsequent Li intercalation into the cathode. As the number of available Li accommodation sites dwindles in the NCM particle near surface regions, the capacity is reduced, and full charging is prevented.

In our opinion, the study by Chae et al. is important because this article is a prime example of how using combinatorial microscopy techniques having similar length-scale resolutions but complementary strengths, can explain the behavior of complex, difficult to characterize material systems, such as phase transformations in these battery electrode materials. All microscopy techniques have strengths and shortcomings, but workflows exist that can exploit their inherent complementarity. For battery materials research, APT is a superior technique to reliably quantify Li concentrations with nanoscale resolution, albeit not without experimental challenges, while (S)TEM techniques are better suited for nano- to atomic-scale structural and local valence state identification. The combination of these techniques reveals a direct correlation of the Li loss in NCM cathodes to local structural disorder and phase transformations that limit the capacity of Li-ion batteries. What’s next? These insights demonstrate possible routes for improving the lifetime of batteries via two possible ideas: (i) adjust the material’s composition to limit the migration of TMs into anti-sites, which is related to the energetics of site-occupancy and hence may be calculable by ab initio techniques; (ii) the cores of the cathode particles remain unchanged, meaning that the Li therein is ‘lost’ or rather unexploited, and as such, designing the grain architecture may help use these unused ions. We hope battery experts seize on these results and integrate them into the design of next generation batteries.
