# Author Correction: Correlating advanced microscopies reveals atomic-scale mechanisms limiting lithium-ion battery lifetime

**DOI:** 10.1038/s41467-021-24594-8

**Published:** 2021-07-08

**Authors:** Baptiste Gault, Jonathan D. Poplawsky

**Affiliations:** 1 Max-Planck-Institut für Eisenforschung, Düsseldorf, Germany; 2 Department of Materials, Royal School of Mines, Imperial College, London, UK; 3 Center for Nanophase Materials Sciences, Oak Ridge National Laboratory, Oak Ridge, TN USA

**Keywords:** Energy, Batteries, Structural properties, Characterization and analytical techniques

Correction to: *Nature Communications* 10.1038/s41467-021-24121-9, published online 18 June 2021.

The original version of this Article omitted a reference to previous work in “Chae, B. G. et al. Evolution and expansion of Li concentration gradient during charge–discharge cycling. *Nat Commun* **12**, 3814 (2021)”. This has been added as reference [14] at “Chae et al. combined... [14]”. This has been corrected in the PDF and HTML versions of the Article.

